# Artificial intelligence awareness, usage, and readiness among fellows of the West African college of physicians

**DOI:** 10.1186/s12909-026-10063-7

**Published:** 2026-08-03

**Authors:** Manmak Mamven, Obinna O Oleribe, Uduak Offiong, George Acquah, Olufemi O. Ogunrinde, Abubakar Haruna, Victor Ansa, Benjamin S.C. Uzochukwu, Simon D Taylor-Robinson

**Affiliations:** 1 Department of Medicine, Faculty of Clinical Sciences, College of Health Sciences, University of Abuja, Gwagwalada, Abuja Nigeria; 2 School of Public Health and Health Sciences, College of Health, Human Services and Nursing, California State University, Dominguez Hills, Carson, CA USA; 3 Department of Community health, Korlebu Teaching Hospital, Accra, Ghana; 4 Department of Paediatrics, College of Health Sciences, Faculty of Clinical Sciences, Ahmadu Bello University, Zaria, Nigeria; 5 Department of Internal Medicine, Faculty of Clinical Sciences, College of Medical Sciences, University of Calabar, Calabar, Nigeria; 6 Department of Community Medicine, College of Medicine, University of Nigeria Nsukka, Enugu, Nigeria; 7 Department of Surgery and Cancer, St Mary’s Campus, Imperial College London, London, UK

**Keywords:** Africa South of the Sahara, Artificial Intelligence, Developing Countries, Medical Education, West African College of Physicians

## Abstract

**Background:**

Artificial intelligence (AI) is transforming healthcare globally, but its adoption remains limited in low- and middle-income countries, such as those in West Africa. Physician educators are central to AI uptake, making their awareness and readiness crucial. This study assessed AI awareness, usage, and readiness among physicians at the West African College of Physicians and provides a roadmap for institutional digital transformation in the region.

**Methods:**

A cross-sectional survey was conducted among 143 trainers who were recruited during a workshop at the organisation’s annual meeting. Data were collected using a structured self-administered Google Forms questionnaire with multiple-choice and Likert-scale questions that assessed awareness, usage, challenges, and factors associated with the frequency of AI usage. The data were analysed using SPSS version 27.

**Results:**

The response rate was 55%, and most participants practised medicine in northern Nigeria (50.5%). While 75% reported some familiarity with AI concepts, only 9.8% were very familiar. About 36% were aware of AI tools for medical education, with ChatGPT being the most recognized. Frequent AI use was observed in 19%, mainly for research, with (55%) using it in their roles as educators. Only 35% had access to AI tools, and 23% had received training on AI. Although 98% believed that AI could improve medical education, and 90% supported its integration, nearly all (99%) preferred AI as a supplement rather than a replacement. Key barriers and concerns to adoption and access were lack of training (63.4%), unavailability of tools (51.7%), poor internet connectivity (51%), and ethical concerns. Frequent AI use was independently associated with having additional MSc or PhD qualifications (OR = 3.7, 95% CI: 1.28–10.96, *P* = 0.016), and AI training attendance. (OR = 2.79, 95% CI: 1.01–7.72, *P* = 0.048).

**Conclusion:**

Despite strong interest and perceived value, AI adoption among WACP fellows and instructors is low due to limited training and access. Efforts to integrate AI into medical education and practice should be led by the WACP through AI policy development and implementation to bridge the AI gap and prepare physicians for digital healthcare.

**Supplementary Information:**

The online version contains supplementary material available at https://doi.org/10.1186/s12909-026-10063-7.

## Introduction

Rapid advancements in artificial intelligence (AI) have revolutionised global health care and presented several key opportunities [1–5]. Contemporary health care delivery relies on a synergy between clinical practice and medical education (ME) to improve patient outcomes, making it essential to assess how medical leaders engage with emerging technologies. The incorporation of AI across clinical practice can transform decision-making through precision diagnostics, decision support, and predictive analytics for personalised care. In ME, AI is transforming training through adaptive learning platforms, algorithmic simulations, and data-driven competency assessments [1–4]. However, the successful integration of AI into healthcare depends not only on technical availability but also on the awareness, acceptance, and readiness of physicians who are the primary end users and gatekeepers of these innovations [6, 7].

Studies assessing healthcare professionals’ knowledge, attitudes, and practices regarding AI have mixed findings, with physicians in high-income countries generally expressing optimism about its ability to improve patient care and healthcare efficiency [8–10]. However, significant concerns and challenges regarding the ethical implications of AI and its potential impact on the professional roles of these physicians have been observed [8–12]. These challenges are often amplified in low- and middle-income countries (LMICs), particularly in sub-Saharan Africa (SSA), where evidence of AI readiness and adoption remains low despite growing digital health ambitions [13–20]. In medical institutions across SSA, implementation is further hindered by constraints such as infrastructure, unreliable internet services, inadequate AI-specific training, the high cost of proprietary platforms, and the absence of comprehensive regulatory frameworks to guide its use [11–13, 21].

Furthermore, West Africa faces a profound burden of diseases amidst a persistent shortage of specialist physicians, creating a critical opportunity for AI-enabled solutions to strengthen healthcare [22]. However, while many countries in SSA have prioritised digital health and experienced rapid technological growth, the adoption of AI into clinical practice and ME remains in its nascent stages [13, 18–21].

Central to the ME landscape is the West African College of Physicians (WACP), a professional organisation whose mandate is to provide postgraduate specialist training, accredit training institutions, and promote excellence in healthcare across West Africa [23]. Trainees are licenced medical doctors who have undergone rigorous, multi-year clinical training in various specialities. Operating in 14 countries and with more than 6000 fellows, the WACP represents the largest network of specialist physicians in the subregion [23]. In addition to being clinicians, WACP fellows are also educators, researchers, policymakers, and leaders who will influence the future integration of AI within healthcare systems. Their level of preparedness for AI adoption may therefore serve as an important indicator of the region’s capacity to implement and govern digital health innovations at scale.

Despite growing global discourse in AI and recommendations to incorporate competencies into ME and specialist training from organisations such as the World Medical Association (WMA) and the Standing Committee of European Doctors (CPME) [24, 25] to incorporate competencies into ME and specialist training, empirical data on AI awareness, utilisation, and readiness among physicians in SSA remain limited [13, 18, 21, 26]. While AI has immense potential for healthcare in West Africa, its systemic adoption depends on the readiness of the region’s senior medical leadership. In SSA, there is a high general awareness of digital health innovations among medical leaders but relatively low clinical utilisation, which signifies a disconnect driven by infrastructural, educational, and policy gaps [20–22]. Existing digital integration frameworks are largely modelled after high-income settings, which fail to address the unique challenges faced by medical institutions in this region [17]. As a leading body for postgraduate medical training in West Africa, WACP plays a critical role in shaping regional clinical practice and ME, yet data concerning AI readiness among its leaders remain limited; to our knowledge, this study represents one of the first assessments of its kind for this group. Given the risk of AI adoption without adequate training or institutional oversight, understanding the baseline readiness of this specific group is essential for developing context-specific training programs and regional ethical policies [22]. Therefore, this study leverages data from WACP fellows attending the October 2024 AGSM in Abuja to quantify the awareness, utilisation, and readiness gap and establish an empirical foundation for the curriculum updates and institutional reforms needed to safely drive responsible AI integration.

## Methods

### Study design

This was a cross-sectional internet-based survey using a structured self-administered questionnaire conducted among attendees of the conference workshop.

### Study setting and context

The workshop was held in Abuja during the 2024 Annual General and Scientific Meeting (AGSM) of WACP. Attendees were mostly fellows in the WACP.

### Study population

The study participants were WACP fellows from institutions in West Africa. A convenience sampling strategy was utilised, drawing from the cohort of healthcare leaders and medical educators who were actively registered for and attending the specific institutional workshop. Potential participants were formally invited to take part in the study during the plenary sessions of the workshop, where the research objectives were presented, and questionnaires were distributed either physically or via a secure digital link. Of the 256 eligible trainers who participated in the workshop, 143 completed and returned the evaluation questionnaire, yielding a robust response rate of 55.9%. About half the participants (50%) practised in Northern Nigerian states, followed by 35.6% from the Southern Nigerian states, while 14% were from non-Nigerian states. The majority were aged between 51 and 60 (32.2%), followed closely by those aged 41 to 50 (30.8%). The commonest highest qualification was the WACP Fellowship only (53.5%). Additional academic qualifications (Fellowship plus PhD, Fellowship/MD/PhD, Fellowship plus MD only) were held by relatively few participants. The dominant faculty was Internal Medicine (43%), followed by Community Health (19.6%) and Paediatrics (18.2%). Almost half of the respondents had 16 or more years’ experience in ME (43%) (supplementary Table A).

### Data collection

We used a structured self-administered online questionnaire that the authors specifically developed for this study to collect data on participant demographics (e.g., age, specialty, years of practice, area of practice), awareness, familiarity, readiness for, and use of AI tools in ME, frequency of AI tool use, and perceptions of AI tools and its benefits, and challenges. The survey instrument was reviewed by experts in ME and AI ethics to ensure its appropriateness, accuracy, and clarity. The survey questionnaire was administered through a secure online Google platform (see supplementary file). Based on the attendee list, the questionnaire was also distributed at the workshop and sent by the attendees through email and WhatsApp messages. Data collection was extended for a period beyond the conference to ensure robust collection. The data collection spanned from October 2024 to January 2025. Participants accessed the survey using their personal devices (e.g., smartphones, laptops, or tablets). We ensured that the questionnaire only allowed one-time access to prevent multiple completions by individual participants. Responses were collected anonymously, and no personally identifiable information was collected.

### Study tool

The research questionnaire was structured into domains to assess the AI awareness, usage, and readiness in ME among WACP fellows. Awareness was defined as the foundational knowledge of AI’s existence or a practical understanding of its applications based on experience [27]. Self-reported familiarity with basic AI concepts and their applications in ME was measured on a 4-point Likert scale ranging from not familiar to very familiar. We also assessed the identification of specific AI tools designed for ME in the domain. Usage was defined as the frequency of AI tool use and was assessed subjectively based on a 5- point Likert scale (never, rarely, sometimes, often, very often). Information on the primary purpose of using AI in their role as educators and what respondents considered to be the most promising applications of AI in ME were also collected. Participants’ comfort levels with the AI were assessed on a 5-point Likert scale (1 is very uncomfortable to 5 is very comfortable). Individual readiness evaluated the immediate infrastructure and attitudes necessary for adoption and included **s**elf-reported access to high-speed internet (yes/no), perceived utility, such as belief in AI’s potential to improve the quality of ME within the WACP, the conviction that AI tools should be integrated to enhance clinical training (Likert scale from strongly disagree to strongly agree), and the perspective on whether AI tools should function as supplements to or replacements for human functions in medical training. Institutional readiness referred to an assessment of the organisational environment of the WACP and its affiliated hospitals. This dimension encompassed the degree to which AI tools were readily available, perceptions of whether current WACP curricula and hospital settings are conducive to support AI integration, and the belief that WACP should provide more financial resources and technical support for the use of AI tools in ME and participation in specialised workshops on training sessions specifically focused on AI. Additionally, it assessed familiarity with WACP-specific guidelines and broader government regulations on AI in ME. All the responses required a yes or no answer.

### Data analysis

Data were imported from Google Forms and analysed using SPSS version 27. The data were presented as frequencies, percentages, means, and standard deviations. The factors influencing the use of AI were examined using ordinal logistic regression (OLR). The outcome variable, the frequency of use of AI, was measured on an ordinal scale and was merged into three categories (i.e., never/rarely, sometimes, and frequently) and both bivariate and multivariable OLR were performed. Variables were selected for the multivariable OLR model if they had a P value < 0.05 in the bivariate analysis or were identified in previous studies as significant predictors. Three models were evaluated for best fit, and the model with the significantly lower Akaike information criterion values is presented. The level of significance for each test was set at *P* < 0.05.

### Ethical considerations

Ethical clearance was obtained from the University of Abuja Human Research and Ethics Committee (UATH/HREC/PR/2024/010/198). The study was conducted under the guidelines of the ethical principles of autonomy, beneficence, non-maleficence, and justice, and the ethical guidelines of the National Human Research Ethics Committee (NHREC) and the Helsinki Declaration of 1975. All participants provided informed consent by clicking on the survey link and agreeing to participate. Participation was voluntary and collected data was kept confidential. Individual responses were not linked to any identifying information.

## Results

This study assessed the awareness, usage, and institutional readiness of AI among fellows of the WACP who attended the AGSM in October 2024 in Nigeria. The findings reveal that, while conceptual familiarity of AI’s medical potential was high (84%) actual clinical adoption remained low. The study contributes an empirical baseline on AI metrics within WACP which fills a critical geographical gap in global health informatics and provides data-driven blueprints to reform the WACP curriculum to employ digital health technologies.

### Awareness, familiarity, and use of AI tools

Table 1 displays the responses of participants on their knowledge and familiarity with basic concepts of AI tools in ME. While 75% reported some familiarity with AI concepts in medicine, only a small fraction 9.8% were very familiar. Awareness of specifically designed AI tools for ME was lower at 36%, with ChatGPT as the most recognized tool. Ready availability of AI tools was reported by only 35% of respondents, while most (98%) agreed that AI has the potential to improve ME quality and a strong majority (90%) agreed or strongly agreed that AI tools should be integrated into ME. Almost all participants (99%) preferred AI tools to supplement and not to replace human educators. However, AI tool usage frequency varied with ‘sometimes’ being the most common response (41%).

**Table 1:** Awareness, familiarity, and use of AI tools for medical education

Description	Frequency	Percent
Familiarity with the basic concepts and applications of AI in medicine (*n* = 143)		
Not familiar at all	21	14.7
Somewhat familiar	64	44.8
Moderately familiar	44	30.8
Very familiar	14	9.8
Awareness of AI tools specifically designed for medical education (*n* = 143)		
No	92	64.3
Yes	51	35.7
Known AI Tools in medical education(*n* = 143)		
ChatGPT	10	7.0
ChatGPT + Other	14	9.8
CoPilot	1	0.7
Grammarly	1	0.7
Multiple (others) *	8	5.6
Others	10	7.0
None	99	69.2
Ready availability of AI tool (*n* = 105)		
No	68	64.8
Yes	37	35.2
Frequency of use or encountering of AI tools in medical education or practice (*n* = 143)		
Never	18	12.6
Rarely	39	27.3
Sometimes	59	41.3
Often	16	11.2
Very often	11	7.7
AI potential to improve the quality of medical education in the WACP (*n* = 143)		
No	3	2.1
Yes	140	97.9
AI tools should be integrated into ME to enhance learning and training for future physicians (*n* = 143)		
Strongly Disagree	2	1.4
Disagree	0	0.0
Neutral	11	7.7
Agree	72	50.3
Strongly Agree	58	40.6
AI tools can supplement or replace humans in medical education (*n* = 143)		
Replace	2	1.4
Supplement	141	98.6

Others * e.g. InSimu, Lecturio simulation app, DynaMedex, Deep learning, Mendeley reference manager, Data analysis tools, quill box, Robotic learning, Me chimes, Aitor, Study bots, Chat labs

### Participants training and attitude to AI

**Fig. 1:**
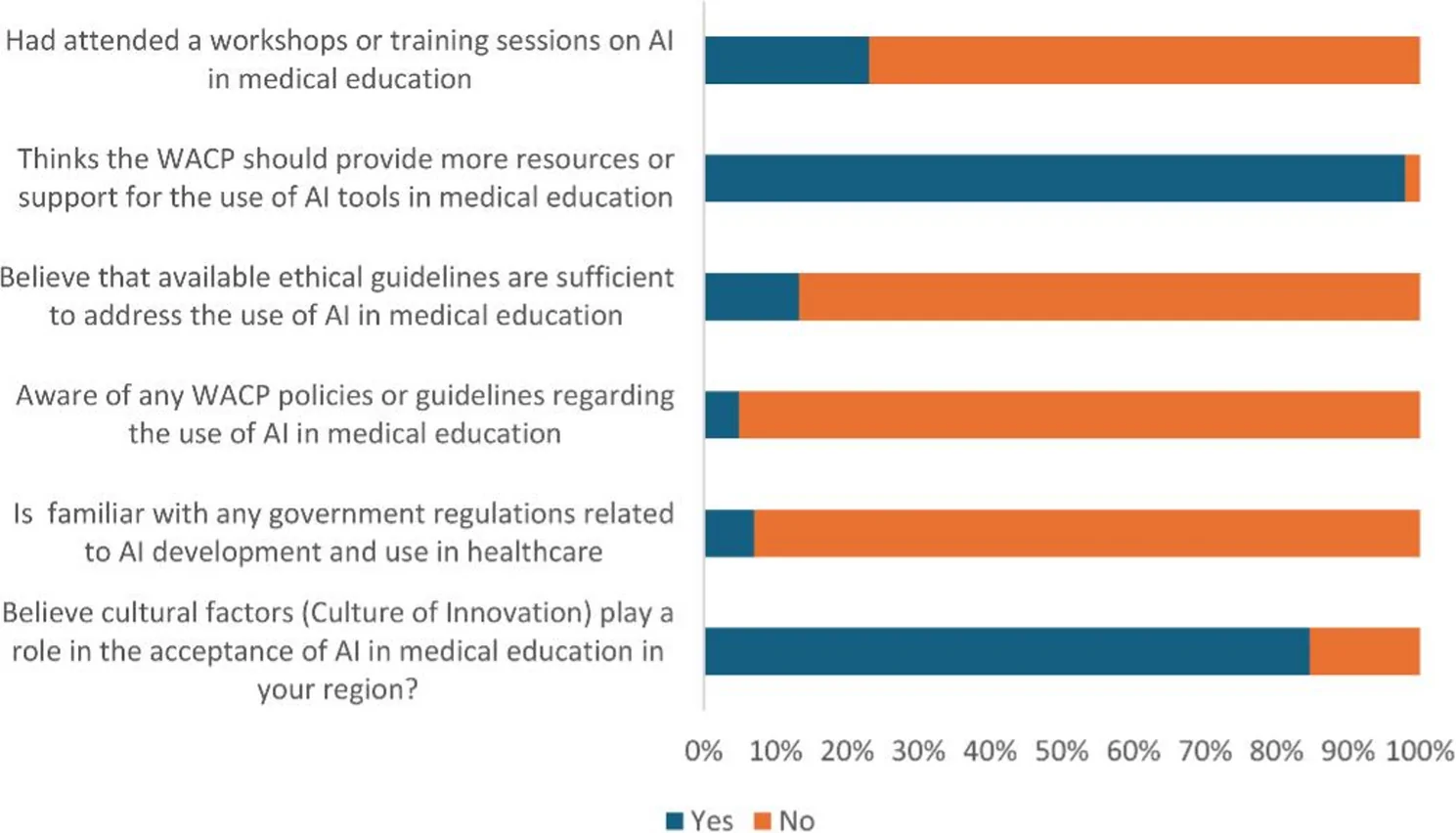
Participants training and attitude to AI in medical education

Figure 1 displays responses to participants training and attitude to AI in ME. About a quarter (23%) had attended a training session on AI in ME. The overwhelming majority (98%) believed the WACP should provide resources or support for AI tools use in ME. Only 13% of respondents believed available ethical guidelines are sufficient to address the use of AI in ME. Only 5% were aware of any WACP policies or guidelines on the use of AI in ME. Again, few respondents (7%) were familiar with any government regulations on AI development and use in healthcare. The majority believed that cultural factors play a role in the acceptance of AI in ME in Nigeria and across the West African region (Fig. 1).

### Uses of AI and users comfort levels

The distribution of primary purpose of use and subjective comfort levels is presented in Table 2. Most of the participants reported research (55%), curriculum development (20%) and trainee assessment (13%) as the primary purpose for using AI tools as educators. Several respondents (35%) reported feeling very comfortable or comfortable while using AI tools in ME with a smaller proportion reporting feeling uncomfortable/very uncomfortable (24%), and 79% thought that the most promising applications of AI in ME were the improved access to learning resources (Table 2).

**Table 2:** Uses of AI and users comfort levels

Primary purpose for using AI tools in your role as an educator (*n* = 143)		
	Frequency	Percent
Administrative tasks	9	6.3
Curriculum development	29	20.3
Research	78	54.5
Trainee assessment	19	13.3
Teaching and assessment	2	1.4
All the above	1	0.7
None	5	3.5

Comfort level while using AI tools in medical practice or education (*n* = 143)		
	Frequency	Percent
Very uncomfortable (1)	11	7.7
Uncomfortable (2)	23	16.1
Neutral (3)	58	40.6
Comfortable (4)	27	18.9
Very comfortable (5)	24	16.8

Most promising applications of AI in ME (*n* = 143)		(n=freq/143)*
	Frequency	Percent
Improve access to learning resources	112	78.6
Data analysis and research	86	61.4
Personalized learning	87	61.4
Automated assessment and feedback	68	48.3

*% frequency /143

### Challenges and concerns in using AI tools

**Fig. 2:**
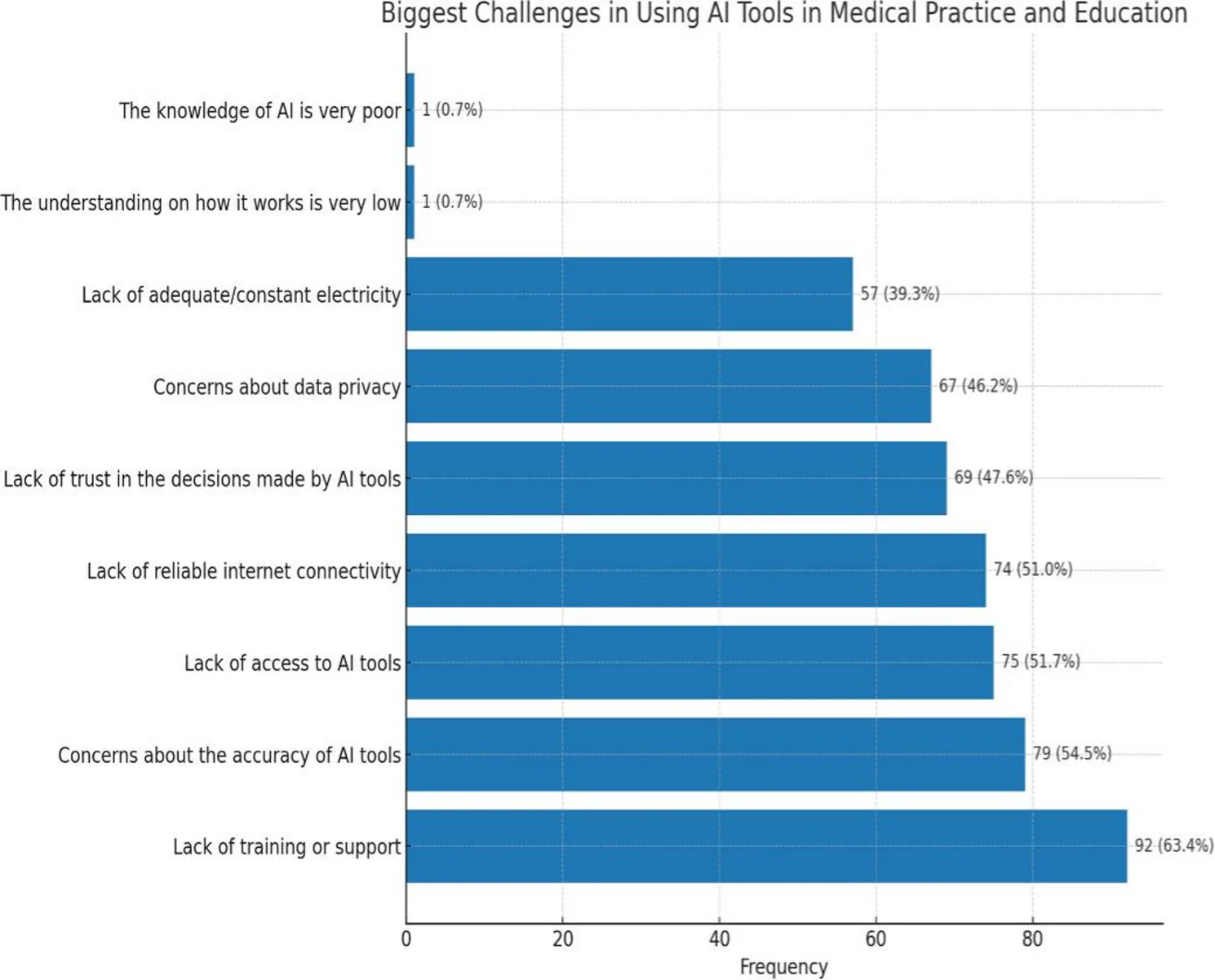
Biggest challenges faced in using AI tools in medical practice and education

The biggest challenges and main concerns faced in using AI tools in ME as reported by the respondents were the lack of training or support (63.4%), followed by concerns about the accuracy of the tools (54.5%). Other challenges mentioned include the lack of access to the tools, lack of internet connectivity, lack of trust in the tool, lack of data privacy, poor understanding of how AI works, and the inadequate knowledge of AI. (Fig. 2)

**Fig. 3:**
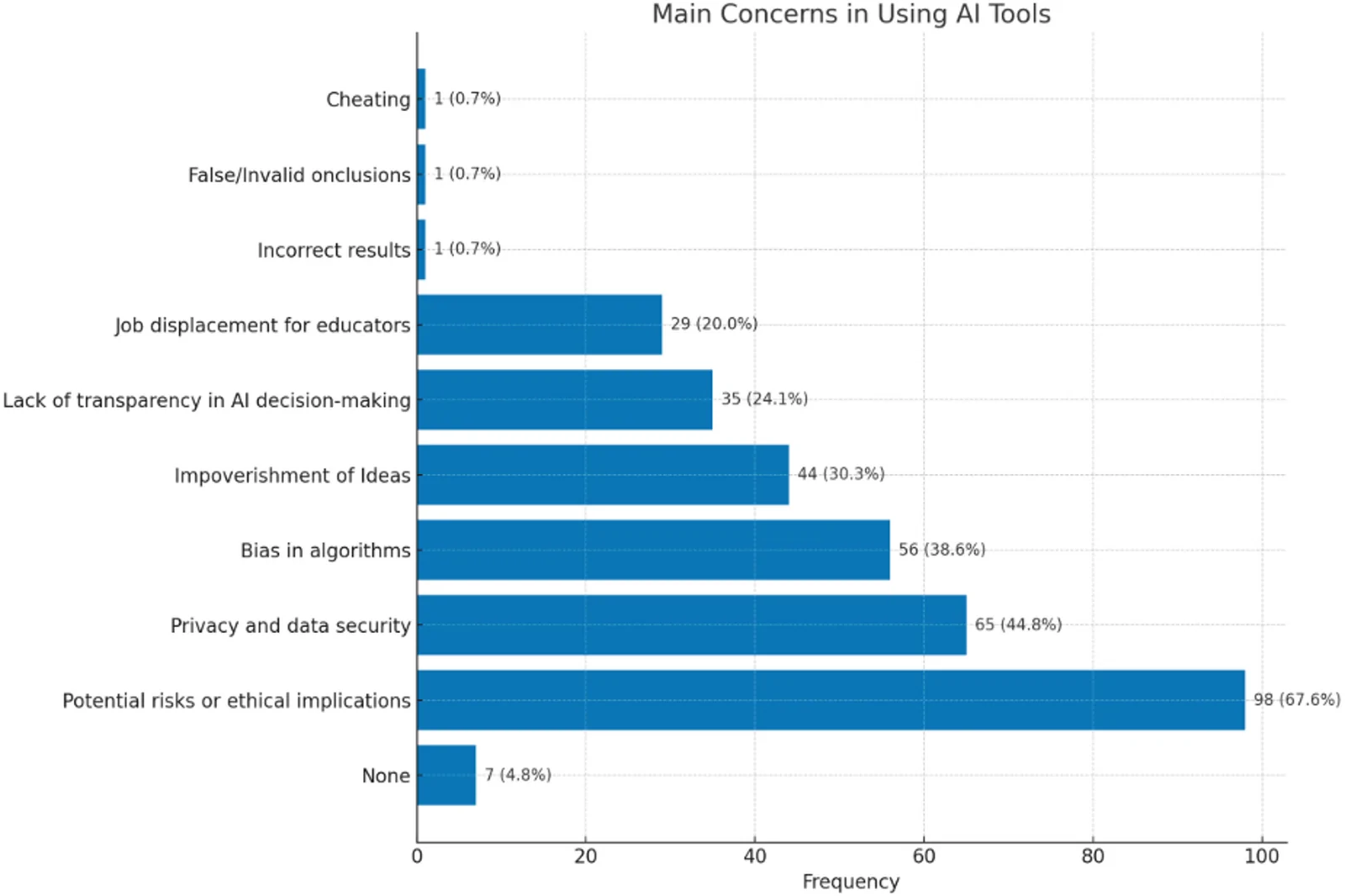
Main concerns in using AI tools

In Fig. 3, participants mentioned potential risk or ethical concerns (68%) as a major concern, followed by the privacy and security of the data (45%), bias in the algorithm (39%), impoverishment of ideas (30%), lack of transparency (24%), and job displacement (20%).

### Frequency of using or encountering AI tools

Table 3 displays the distribution of frequency of encountering or using AI tools in ME. Frequent AI use was more in respondents with additional qualification MSc/MPH (27.8%) or PhD/MD (23.3%), than in those with a Fellowship alone (13.2%). About 86% with very familiar response and 47% of comfortable trainers reported frequent AI use. Conversely, barriers such as a lack of awareness of AI tools specific for AI in ME and limited tool availability dropped frequent use rates to, 8.7% and 8.8%, respectively. Importantly, participation in training on AI increased the frequency of use from 15% to 30%. Respondents with trust in the decisions made by AI tools used AI tools more frequently than those with lack of trust (21% vs. 16.4%).

**Table 3:** Frequency of using or encountering AI tools in medical practice or education

Factors	Total	Never/Rarely *n* (%)	Sometimes *n*(%)	Frequently *n*(%)
Age group (*n* = 143)				
30 or less	1			
31–40 years	24	11(45.8)	10 (41.7)	3 (12.5)
41–50 years	44	16 (36.4)	19 (43.2)	9 (20.5)
51–60 years	46	16 (34.8)	19 (41.3)	11(23.9)
> 60 years	28	14 (50.0)	10 (35.7)	4 (14.3)
Area of practice: Geographic distribution (*n* = 143)				
Northern Nigerian states	72	29(40.3)	29(40.3)	14(19.4)
Southern Nigerian states	51	21(41.2)	21(41.2)	9(17.7)
Other non-Nigerian/West African states*	20	7(35.0)	9(45.0)	4(20.0)
Highest Qualification (*n* = 142)				
WACP Fellowship only	76	40 (52.6)	26 (34.2)	10 (13.2)
+ MSc or MPH	36	9 (25.0)	17 (47.2)	10 (27.8)
+ PhD/MD	30	7 (23.3)	16 (53.3)	7 (23.3)
Faculty in WACP				
Community Health	28	8(28.6)	11(39.3)	9(32.1)
Family Medicine	17	9(52.9)	3(17.7)	5(29.4)
Internal Medicine	61	25(40.9)	31(50.8)	5(8.2)
Paediatrics	27	11(40.7)	11(40.7)	5(18.5)
Psychiatry/Lab Medicine	6	3(50.0)	2(33.3)	1(16.7)
Familiarity with the basic concepts and applications of AI in medicine (*n* = 143)				
Not familiar at all	21	16(76.2)	4(19.1)	1(4.8)
Somewhat familiar	64	31(48.4)	29(45.3)	4(6.3)
Moderately familiar	44	9(20.5)	25(56.8)	10(22.7)
Very familiar	14	1(7.1)	1(7.1)	12(85.7)
Awareness of any AI tools specifically designed for medical education? (*n* = 143)				
No	92	45(48.9)	39(42.4)	8(8.7)
Yes	51	12(23.5)	20(39.2)	19(37.3)
No access to use of AI tools (*n* = 143)				
No	66	23(34.9)	26(39.4)	17(25.8)
Yes	77	34(44.2)	33(42.9)	10(12.9)
Are these tools readily available to you? (*n* = 105)				
No	68	35(51.8)	27(39.7)	6(8.8)
Yes	37	3(8.1)	16(43.2)	18(48.7)
Comfort level while using AI tools in medical practice or education (143)				
Very /uncomfortable	34	24(70.6)	9(26.5)	1(2.9)
neutral	58	24(41.4)	32 (55.2)	2(3.5)
comfortable/very comfortable	51	9(17.7)	18(35.3)	24(47.1)
Attended any workshops or training sessions on AI in medical education? (*n* = 143)				
No	110	53(48.2)	40(36.4)	17(15.5)
Yes	33	4(12.1)	19(57.6)	10(30.3)
Lack of reliable internet connectivity(*n* = 143)				
No	70	28(40.0)	30(42.9)	12(17.1)
Yes	73	29(39.7)	29(39.7)	15(20.6)
Lack of trust in the decisions made by AI tools(*n* = 143)				
No	76	33(43.4)	27(35.5)	16 (21.1)
Yes	67	24 (35.8)	32(47.8)	11(16.4)

Table 4 shows the bivariate and multivariable OLR analysis of factors associated with frequency of using AI tools in ME. Independent factors associated with frequent usage of AI tools were additional MSc or MPH qualification (OR, 3.7 ,95% CI; 1.28–10.96, *P* = 0.016). Participants who reported neutral comfort level with AI tools use had significantly higher odds of usage than those who were very uncomfortable/uncomfortable (OR 6.2, 95%CI;1.60-23.82, *P*<0.008). while participants who attended any workshops or training sessions on AI had significantly higher odds of frequent usage compared to those who did not (OR 2.79, 95%CI; 1.01–7.72, *P*<0.048).

**Table 4:** Factors associated with frequency of using AI tools in medical education or practice

Factors	CrOR (95% CI)	*P*-value	aOR^*^ (95% CI)	*P*-value
Age group				
31–40 years	1(ref)		**--**	
41–50 years	1.3 (0.49-3.25)	0.63		
51–60 years	1.3 (0.49-3.18)	0.64		
> 60 years	0.80 (0.28-2.29)	0.68		
Area of practice: Geographic distribution				
Northern Nigerian states	0.81 (0.32-2.05)	0.66	**--**	
Southern Nigerian states	0.81 (0.31-2.14)	0.68		
Others Non-Nigerian/W.African states*	1(ref)			
Highest qualification				
WACP Fellowship only	1(ref)	.	1	
+ MSc or MPH	2.3(1.11–4.91)	0.026	3.7 (1.28–10.96)	0.016
+ PhD/MD	2.8 (1.24–6.26)	0.013	2.1 (0.75-5.62)	0.16
Faculty in WACP				
Community Health	1(ref)		**--**	
Family Medicine	0.4 (0.14-1.24)	0.11		
Internal Medicine	1.03 (0.45-2.33)	0.95		
Paediatrics	0.8 (0.311 − 2.15)	0.68		
Psychiatry/Lab Medicine	0.6 (0.11-3.02)	0.51		
Familiarity with the basic concepts and applications of AI in medicine				
Not familiar	1(ref)		1	
Somewhat familiar	4.1(1.34–12.62)	0.013	2.7 (0.59-12.43)	0.2
Moderately familiar	9.5(2.96–30.52)	0	2.3 (0.46-11.77)	0.31
Very familiar	4.4(1.23–15.94)	0.02	1.6 (0.24-10.66)	0.62
Comfort level while using AI tools in medical practice or education				
Uncomfortable	1(ref)		1	
Neutral	4.3(1.73–10.74)	0.002	6.2(1.60-23.82)	0.008
Comfortable	4.2(1.72–10.07)	0.002	3.0 (0.67-13.47)	0.15
Access to use of AI tools				
No	1(ref)		**--**	
Yes	0.89(0.48-1.64)	0.71		
Attended any workshops or training sessions on AI in medical education				
No	1(ref)		1	
Yes	3.17 (1.51–6.67)	0.002	2.8 (1.01–7.72)	0.048
Awareness of AI tools specifically designed for medical education				
No	1(ref)		**--**	
Yes	1.54 (0.82-2.88)	0.18		
Are AI tools readily available to you?				
No	1(ref)		1	
Yes	2.49 (1.19–5.21)	0.02	1.2 (0.52-2.56)	0.73
Lack of reliable internet connectivity				
No	1(ref)		**--**	
Yes	0.9(0.51-1.74)	0.85		
Lack of trust in the decisions made by AI tools				
No	1(ref)		**--**	
Yes	1.5(0.82-2.81)	0.19		

For model 1 Akaike information criterion (AIC = 225.71) and Bayesian Information Criterion (BIC = 291.584) For model 2^*^ (AIC = 216.86) and (BIC = 251.23) values. suggesting that model 2 is more robust and efficient in estimating the predictors of frequent AI use. *CrOR *Crude odds ratio, *aOR* Adjusted odds ratio

## Discussion

This study evaluated the awareness, utilisation, and readiness for AI among physicians within the WACP and the factors driving its adoption. The findings show that while there is some awareness, there is still a critical readiness gap, especially in institutional infrastructure deficits and systemic anxieties over data privacy and algorithmic bias. This study offers a pioneering contribution as the first sub-regional assessment of AI in such a cohort and provides a critical baseline for understanding how WACP leaders perceive and interact with emerging digital technologies such as AI.

### Awareness and knowledge

Our results revealed that while 84% of the participants reported varying degrees of familiarity with the basic concepts and applications of AI tools in medicine, nearly 65% of the participants were entirely unaware of any AI tool specific to ME. This aligns with the findings of Oluwadiya et al., who reported low AI knowledge among medical educators in southern Nigeria [13]. Similarly, authors in other regions of the world highlighted poor basic AI knowledge and low familiarity among medical doctors and educators in diverse settings [28–33]. In contrast, some regional studies reported higher general AI knowledge (68.8%) among clinicians [20]. These variations in knowledge and awareness of AI in ME and medical practice across countries, especially in LMICs, occur due to a complex interplay of poor infrastructure access, lack of AI training, and the timing of studies. Furthermore, the perceived knowledge of AI may reflect a lack of distinction between general-purpose AI tools and more technical tools.

### Usage patterns

Despite the low usage rate of 20%, an overwhelming 98% of the respondents expressed support for AI integration in ME. This mirrors findings from several other authors who also observed low AI usage among medical educators in diverse settings [13, 28–30]. Furthermore, our findings align with observations in India and other developing nations, where there is high interest in AI despite low basic knowledge and practical usage among physicians [18, 31, 32]. In the United States, a growing majority of physicians are recognising the benefits of AI and feel more excited than concerned about it (up to 30% in 2023) [8]. In contrast, while 46.8% of the participants in a Nigerian study had higher utilisation than our cohort did (20%), only 54.4% of the participants had positive attitudes towards the technology [20]. Our findings suggest that WACP instructors are psychologically prepared for AI adoption but are hindered by many challenges. Nevertheless, strong interest in AI lays the groundwork for developmental pathways.

The most common AI tools identified by our respondents were general purpose AI tools such as ChatGPT and Copilot reflecting the current practical reality of AI exposure among instructors in the region. These general-purpose AI tools often serve as the initial entry point for medical pedagogy because of their accessibility, whereas specialized ME AI tools (e.g., OSCE simulators or adaptive platforms such as Aquifer) are cost prohibitive for many African institutions.

### Barriers and challenges

Most respondents (65%) cited the unavailability of specific AI tools and lack of formal training as major setbacks. These barriers are a recurring theme in LMICs; [13, 14, 30, 33, 34] for instance, a report on Vietnam identified inadequate digital platforms and low faculty readiness as universal barriers to moving beyond fragmented AI pilots [30]. Ethical concerns were also a critical issue. While only 20% of our participants feared job displacement, there was a high level of concern regarding algorithmic bias and data privacy in the use of AI. Perspectives on AI-driven job security were divided. Although some reports indicate concerns regarding job loss [9–12], a significant proportion of physicians perceive AI as a collaborative asset [35]. These clinicians welcome AI to automate routine tasks, allowing for greater focus on direct patient care [35]. AI models are trained on Caucasian datasets and may introduce sociocultural biases that are unrepresentative of African populations [36]. To address these concerns, the WACP must align with the WHO AI ethics guidelines for LMICs, which advocate for strategic partnerships in the co-development of models using local African data to ensure that AI serves as a culturally representative, safe, and equitable supplement to human expertise [37].

The challenges observed led 90% of the respondents to express the need for the college to provide training and resources for AI integration.

### Facilitators and readiness

Resource availability, specific skill development, and psychological comfort were the drivers of readiness, which highlights the need for strategies beyond mere awareness and exposure. Notably, those with additional academic qualifications (e.g., MSc, MPH, PhD, or MD) were more likely to use AI tools compared to those with other WACP fellows. This suggests that research exposure is a strong predictor and may act as a catalyst for digital adoption [10]. We also observed that participants who reported being comfortable or very comfortable with AI tools were more strongly associated with AI tool use (47%) than were those who were very uncomfortable or uncomfortable (3%) (Table 3). Our model showed that individuals with neutral comfort levels had significantly higher odds of using AI than those who were uncomfortable (OR = 6.2; 95% C1: 1.60-23.82, *P*=0.008). This may imply that a shift from discomfort to neutrality represents a critical psychological threshold in digital adoption [38, 39]. Uncomfortable individuals may harbour active resistance due to concerns that serve as a barrier to engagement [39]. On the other hand, those with neutral sentiments likely view AI as a functional utility other than an ethical or technical dilemma [38]. Therefore, addressing initial technical anxiety may be vital for institutional integration.

We acknowledge several limitations in our study. First, because the survey was restricted to workshop attendees in Abuja and had an overrepresentation of certain regions, the results are more reflective of an engaged subset of the college rather than a true cross-section of the regional instructor workforce. Second, social desirability bias may have occurred where participants over-reported their familiarity with or usage of AI. The self-reported metrics used were subjective; thus, the current data provide a baseline of perceived rather than actual proficiency.

Wide confidence intervals in the OLR were obtained, which affects the precision, likely due to the small sample size; thus, the results should be interpreted with caution. The data collection period from October to February was extended to increase participation; thus, compared with earlier respondents, those who responded during a later period may report greater awareness, as they may have been influenced by technological updates and media coverage of AI. Nevertheless, these constraints do not invalidate the results, so the present study till serves as a foundation for future, more targeted multi-centre investigations into AI adoption among physicians in West African colleges.

To ensure the successful integration of AI into the WACP, we recommend that national authorities establish regional policies and clear guidelines for the region that align with international best practices [37, 40]. The college should implement a structured AI literacy curriculum across all levels and train on AI competencies, ethics, and integrity that address algorithmic bias in African populations while setting clear boundaries for AI tool use. Additionally, the WACP should train dedicated validators to oversee AI outputs through strategic partnerships with developers to co-create tools that incorporate African data for diversity and representativeness [40].

## Conclusion

Artificial intelligence has vast potential to improve ME, research, and patient care. Despite the current low awareness, access, and usage of AI tools for ME, WACP instructors were enthusiastic about its potential benefits. Key barriers, such as training, insufficient resources, and ethical concerns, should be addressed to fully leverage AI and prepare future physician educators for the evolving digital healthcare landscape. To integrate AI into ME, the WACP must implement targeted interventions such as regional policy development, curriculum development and updates, and robust training.

## Supplementary Information

Supplementary Material 1.

## Data Availability

The data that support the findings of this study are not openly available due to reasons of sensitivity.The authors will provide the raw data upon request, without any unwarranted delays or restrictions. For additional questions or inquiries, please contact the corresponding author.

## Appendix

Supplementary table 5 displays the characteristics of the participants, The majority were aged between 51 to 60 (32.2%) followed closely by the 41 to 50 age group (30.8%). The commonest highest qualification was the WACP Fellowship only (53.5%). Additional academic qualifications (Fellowship plus PhD, Fellowship/MD/PhD, Fellowship plus MD only) were held by relatively smaller numbers of participants. The dominant faculty was Internal Medicine (43%) followed by Community Health (19.6%) and Paediatrics (18.2%). Almost half of the respondents had 16 or more years’ experience in ME (43%).

**Table 5:** Demographic characteristics of survey respondents

Characteristics	Frequency	Percentage
Area of practice: Geographic distribution (*n* = 143)		
Northern Nigerian states	72	50.3
Southern Nigerian states	51	35.6
Other non-Nigerian/West African countries*	20	14.0
Age at last birthday (*n* = 143)		
30 or less	1	0.7
31–40	24	16.8
41–50	44	30.8
51–60	46	32.2
Above 60	28	19.6
Highest Qualification (*n* = 142)		
WACP Fellowship only	76	53.5
WACP Fellowship + MPH or MSc	36	25.3
WACP Fellowship + PhD	7	4.9
WACP Fellowship /MD/PhD	10	7.0
WACP Fellowship + MD	13	9.2
Faculty in WACP (*n* = 138)		
Community Health	28	19.6
Family Medicine	17	11.9
Internal Medicine	61	42.7
Laboratory Medicine	3	2.1
Paediatrics	26	18.2
Psychiatry	3	2.1
No response	5	3.5
Years of experience in medical education (*n* = 142)		
1–5 years	21	14.7
6–10 years	23	16.1
11–15 years	36	25.2
16 + years	62	43.4

* Benín Republic, Cote d’Ivoire, Ghana, Gambia, Liberia, Sierra Leone, Senegal and Togo. *WACP-west African college of physician*
