# Supplementary material for: Artificial intelligence awareness, usage, and readiness among fellows of the West African college of physicians
Source: BMC Med Educ. 2026 Aug 3;26:1757. doi: 10.1186/s12909-026-10063-7 (PMC13721466; doi:10.1186/s12909-026-10063-7)
Supplement: Supplementary file 1 [file 12909_2026_10063_MOESM1_ESM.pdf]

Assessing the Utilization of Artificial Intelligence (AI) Tools in Medical Education among Fellows of the West African College of Physicians (W.A.C.P).

We invite you to participate in a brief survey about the use and acceptance of AI tools in medical education among WACP Fellows. The College plays a crucial role in training of postgraduate doctors while maintaining the highest standards of medical practice across the region. Understanding how AI tools are being used and perceived by WACP physicians is vital for optimizing medical education and ensuring that these advancements benefit healthcare delivery in West Africa.

Our objective is to determine the current use of AI tools in medical education within the WACP. This survey will take approximately 5-7 mins to complete. By completing and submitting the survey, you are providing consent to participate in the study. Your individual privacy and confidentiality are paramount. All responses will be kept anonymous and will not be linked to any personal information. We ask you to fill it out with as much accuracy and honesty. Please understand your participation is entirely voluntary. Ethical approval for the study was obtained from U.A.T.H H.R.E.C.

Thank you for your time and valuable contribution!

Dr Manmak Mamven,
University of Abuja,
Principal researcher.

* Indicates required question

1. Email *

Untitled Section

Skip to question 2

Demographics

2. **Name of institution where you work** *

____

3. **How old are you (at last birthday)?** *

____

4. **What is your current position? (select all that apply)** *

Check all that apply.

- [ ] Medical School Faculty
- [ ] Residency trainer
- [ ] Residency Program Director
- [ ] Medical Education Administrator
- [ ] Chief medical Director
- [ ] Other: _______________________________

5. **What is your highest level of education?** *

Mark only one oval.

- ( ) FWACP
- ( ) FWACP/MD
- ( ) FWACP/MSc
- ( ) FWACP/PhD
- ( ) FWACP/MD/PHD
- ( ) Other: _______________________________

6. **What is your Faculty in WACP?** *

Mark only one oval.

- Community Health
- Family Medicine
- Internal Medicine
- Laboratory Medicine
- Pediatrics
- Psychiatry
- Other: ____________

7. **How many years of experience do you have in medical education?**

Mark only one oval.

- 1-5 years
- 6-10 years
- 11-15 years
- 16+ years
- Other: ____________

Knowledge of Artificial Intelligence (AI) in Medical Education

8. How familiar are you with the basic concepts and applications of AI in medicine? *

Mark only one oval.

- Not familiar at all
- Somewhat familiar
- Moderately familiar
- Very familiar

9. **Are you aware of any AI tools specifically designed for medical education** *

Mark only one oval.

- Yes
- No

10. **If yes to above question, Can you list any of the AI tools you know?**

11. **Are these tools readily available to you?**

Mark only one oval.

- Yes
- No

12. **What do you consider the most promising applications of AI in medical education?** (Select all that apply) *

Check all that apply.

- [ ] Personalized learning
- [ ] Automated assessment and feedback
- [ ] Improved access to learning resources; Simulation and virtual reality
- [ ] Data analysis and research

Current Use of AI in Medical Education/AI Tool Use and Acceptance

13. **How frequently do you encounter or use AI tools in your medical practice or education?** *

Mark only one oval.

- Never
- Rarely
- Sometimes
- Often
- Very often

14. **Do you believe AI has the potential to improve the quality of medical education in the WACP?**

Mark only one oval.

- Yes
- No

15. **To what extent do you agree AI tools should be integrated into medical education to enhance learning and training for future physicians?** *

Mark only one oval.

- Strongly Agree
- Agree
- Neutral
- Disagree
- Strongly Disagree

16. **Do you believe AI tools can supplement or replace human in medical education?** *

Mark only one oval.

- Supplement
- Replace

17. **What will be your primary purpose for using AI tools in your role as an educator?** *

Mark only one oval.

- Student assessment
- Curriculum development
- Research
- Administrative tasks
- None
- Other: ____________________

18. **How comfortable are you using AI tools in your medical practice or education?** *

Mark only one oval.

Not at all comfortable

1
2
3
4
5

Very comfortable

19. **What are your main concerns in using AI tools?** (Select all that apply) *

Check all that apply.

- [ ] Bias in algorithms
- [ ] Privacy and data security
- [ ] Lack of transparency in AI decision-making
- [ ] potential risks or ethical implications
- [ ] Job displacement for educators
- [ ] None
- [ ] Impoverishment of Ideas
- [ ] Other: _______________

20. **Have you attended any workshops or training sessions on AI in medical education?** *

Mark only one oval.

- ( ) Yes
- ( ) No

21. **Do you think the WACP should provide more resources or support for the use of AI tools in medical education?**

Mark only one oval.

- ( ) Yes
- ( ) No

Challenges and Barriers

22. **What are the biggest challenges you face or will face in using AI tools in your medical practice or education?** (Check all that apply) *

Check all that apply.

- [ ] Lack of access to AI tools
- [ ] Lack of training or support
- [ ] Concerns about data privacy
- [ ] Concerns about the accuracy of AI tools
- [ ] Lack of reliable internet connectivity
- [ ] Lack of adequate/constant electricity
- [ ] Lack of trust in the decisions made by AI tools
- [ ] Other: ______________________

23. **Do you believe available ethical guidelines are sufficient to address the use of AI in medical education?** *

Mark only one oval.

- ( ) Yes
- ( ) No

24. **Are you aware of any WACP policies or guidelines regarding the use of AI in medical education?** *

Mark only one oval.

- ( ) Yes
- ( ) No

25. **Are you familiar with any government regulations related to AI development and use in healthcare?** *

Mark only one oval.

- ( ) Yes
- ( ) No

26. **Do you believe cultural factors (Culture of Innovation) play a role in the acceptance of AI in medical education in your region?** *

Mark only one oval.

- Yes
- No

Thank you for your time and valuable contribution!

This content is neither created nor endorsed by Google.

Google Forms
